# Oral HPV Infection and Sexuality: A Cross-Sectional Study in Women

**DOI:** 10.3390/ijms12063928

**Published:** 2011-06-10

**Authors:** Camille Ragin, Robert Edwards, Margaret Larkins-Pettigrew, Emanuela Taioli, Stacy Eckstein, Natalie Thurman, Jessica Bloome, Nina Markovic

**Affiliations:** 1 Cancer Prevention and Control Program, Fox Chase Cancer Center, PA 19111, USA; 2 Division of Gynecologic Oncology, Magee Women’s Hospital, University of Pittsburgh, Pittsburgh, PA 15260, USA; E-Mails: edwarp@mail.magee.edu (R.E.); drpettigrew@aol.com (M.L.-P.); 3 Institute of Translational Epidemiology, Mt. Sinai Medical Center, NY 10029, USA; E-Mail: taiolema@gmail.com; 4 Department of Epidemiology, University of Pittsburgh, Graduate School of Public Health and the University of Pittsburgh Cancer Institute, PA 15260, USA; E-Mails: ste11@pitt.edu (S.E.); thurman_natalie@yahoo.com (N.T.), ninam@pitt.edu (N.M.); 5 Department of Epidemiology, State University of New York, Downstate Medical Center, NY 11203, USA; E-Mail: jessica.bloome@gmail.com

**Keywords:** sexual behavior, HPV, oral, cervical

## Abstract

Human Papillomavirus (HPV) is the main risk factor for cervical cancers and is associated with close to 36% of oropharyngeal cancers. There is increasing evidence that oral HPV transmission is related to sexual behavior but to our knowledge studies that involve women who have sex with women have not been performed. We examined the prevalence of oral HPV according to sexual behavior among a population-based sample of 118 women and have made some inferences of possible predictors of oral HPV infection. Women were categorized as heterosexual (history of vaginal sex and/or oral sex with males only, *n* = 75), bisexual (history of vaginal sex and oral sex with females, *n* = 32) and other (no history of vaginal sex but oral sex with females [homosexuals], virgins and women with incomplete sexual exposure data, *n* = 11) The prevalence of oral HPV infection was 12/118 (10.2%) for the overall study population and was not significantly different between heterosexual and bisexual women (10.7% (8/75) *vs.* 12.5% (4/32), *p* = 0.784). There was no oral HPV detected among homosexual women, virgins or among women where sexual exposure was unknown. Never smokers were more likely to be oral HPV+ compared to former smokers (Adjusted Odds Ratio (Adj OR) = 0.1, 95% CI, 0.0–1.1) and there was no difference in risk between never smokers and current smokers (Adj OR = 0.7, 95% CI, 0.1–4.6). Twenty-five percent (3/12) of oral HPV+ women had a history of HPV and/or genital warts compared to 9% (10/106) of oral HPV-women (*p* = 0.104). For the women with a history of vaginal sex (*n* = 110), oral HPV status was statistically significantly different according to oral sex exposure (*p* = 0.039). A higher proportion of oral HPV-positive women reported that they had no history of oral sex exposure compared to oral HPV-negative women (4/12, 33% *vs.* 7/98, 8%). The prevalence of cervical HPV infection did not vary between heterosexuals and bisexuals (35.7% (25/70) *vs.* 35.5% (11/31), *p*-value 0.411) and for all other women the cervical HPV prevalence was significantly lower (11.1%, 1/9). Our study suggests that smoking and sexual behavior involving males rather than female partners may be possible predictors of oral HPV infection in women. Further studies with larger sample size are needed to confirm these findings.

## 1. Introduction

Human Papilloma Virus (HPV) is the main risk factor for cervical cancers and is associated with about 25% of head and neck cancers, including close to 36% of oropharyngeal cancers [1,2]. In case-control studies of oral HPV, prevalence among controls ranged from 5.0% to 9.2% [3,4]. Population-based cross-sectional studies have shown widely varying prevalence of HPV in the normal oral mucosa, from 0.6% to 81% [5–16]. One review found a prevalence of oral HPV infection among healthy people to be 13.5% [12], while a study in Finland with 2 years of follow-up found oral HPV prevalence rates fluctuating between 15–27% [13].

Despite evidence of HPV as an etiologic risk factor for head and neck cancer, most notably HPV type 16 [17], there are few population-based studies on mechanisms of HPV transmission. Initial studies have shown smoking, age, and HIV positive serostatus as risk factors [10,18]. A recent review by Termine *et al.* evaluated the prevalence of oral HPV infection in women with cervical HPV infection and performed a meta analysis of the current literature [19]. The pooled prevalence of oral HPV infection was 18.1% (95% CI: 10.3–25.9) and the only significant predictor of oral HPV infection was a younger age at first sexual intercourse and no association was found between oral HPV infection and oral/genital sex. However, the gender of the sexual partner was not considered in these analyses and it is not yet clear whether the relationship between sexual behavior and risk of oral HPV infection differs according to the gender of the sexual partner. Furthermore, there is increasing evidence from case-control studies that oral HPV status may be related to sexual behavior, and particularly oral sexual behavior. Case-control studies of head and neck cancer have shown associations with number of sexual partners, history of oral-genital contact, history of genital warts, and age at first intercourse [4,18]. Males with HPV-positive tumors were also more likely to report a partner with history of abnormal Pap smear or cervical dysplasia [18]. This suggests that sexual behavior risk factors are a proxy for exposure to high risk HPV types. A recent study of risk factors for oral HPV indicated not only number of vaginal and oral sex partners, but also number of kissing partners were associated with oral HPV status [5]. To our knowledge, there are no studies that have evaluated the risk of oral HPV infection based on sexual behaviors by comparing the gender of the sex partners. More data are needed to confirm the main route of oral HPV transmission.

Among women who have sex with women, previous studies have shown cervical HPV prevalence of 13% and 30%, including cases among women with a history of exclusively female sexual partners [20–22]. This indicates sexual transmission of HPV may occur between women. In this study we have examined the prevalence of oral HPV and we have evaluated the sexual behaviors in women in order to identify potential risk factors associated with oral HPV infection.

## 2. Methods

### 2.1. Study Population

This cross-sectional study involves participants who were enrolled in the Epidemiologic Study of HEalth Risk (ESTHER) project (a study of cardiovascular disease focused in comparisons between heterosexual and homosexual women). The ESTHER project’s participants were recruited from participants attending women-focused events, news items and advertisements in women-focused news sources throughout the city of Pittsburgh, Pennsylvania. Following approval by the University of Pittsburgh Biomedical Institutional Review Board (IRB), each ESTHER project participant (*N* = 1043) was contacted by the study coordinator (by mail and a follow up telephone call) and invited to participate in this pilot study and if they agree to do so they were consented prior to initiation of the study. All women were called at least once for recruitment into this study and 88 agreed to participate (self-identified heterosexual women = 52; self-identified homosexual women = 36). All study participants were required to be seen at one clinic visit by trained research staff in the Magee Womens Hospital Clinical & Translational Research Center (CTRC). In addition the study population was enriched with 30 African-American participants who were also enrolled during their routine gynecologic clinic visit at the Magee Womens Hospital.

### 2.2. Sample and Data Collection

During the clinic visit, an oral brushing, mouthwash and a cervical brushing was collected to determine HPV status and the type of HPV present. The oral brush of the tonsils was collected first using 10 strokes (5 on each side) with a sterile nylon bristle cytology brush. This was followed by the mouthwash collection which required gargling 1–2 min while tilting the head back slightly toward the back of the throat. The goal was to gather exfoliated cells from the tonsil area. The results from each of these sampling techniques were combined to provide the oral HPV status. For the 30 African-American women, only a mouthwash sample was collected.

Women who had a Pap smear performed within the previous 12 months prior to the study visit and had the results available were given the option of providing these results at the time of their study visit. Other participants had a Pap smear specimen collected by a study nurse, during their study visit. These Pap smears were read by qualified personnel at Magee Women’s Hospital, Department of Cytology and confirmed by a pathologist. The results of these Pap smears were made available to each study participant at no cost. Women with abnormal Pap test results were referred for follow up with their primary care provider. If the participant did not have their own primary care provider, one was recommended to them. History of abnormal cervical cytology (ASCUS, CIN or cancer) was determined by combining the history collected from questionnaire data with Pap smear results generated at the time of study enrollment and/or 12 months prior to the time of study enrollment. All participants were asked in the questionnaire whether they had a history of abnormal Pap smears (*i.e*., prior to their enrollment in this study). For the participants who answered no to this question, if the Pap smear collected during this study were abnormal (ASCUS, CIN or cancer) they were also classified as having a history of abnormal Pap smears.

Self-reported sexual orientation, age, smoking status, history of previous abnormal cytology diagnoses, history of cervical cancer diagnosis, and sexual behavior (oral and vaginal sexual practices, previous diagnosis of HPV/genital warts, and other sexually transmitted diseases) were obtained for each participant. In this analysis self-reported sexual orientation was not used because it did not correlate with reported sexual behavior patterns. Instead sexual orientation was redefined based on the combination of vaginal and oral sex exposure data and was categorized as heterosexual, bisexual or other (homosexuals, virgins and women with incomplete sexual exposure data). Women with heterosexual exposure were defined as having had vaginal sex and/or oral sex with males only. Women with bisexual exposure had vaginal sex and had oral sex with females and those with homosexual exposure never had vaginal sex and only had oral sex with females.

### 2.3. Laboratory Testing

DNA was extracted from all cervical and oral samples using the Puregene DNA purification kit (Qiagen, Germantown, MD, USA) and HPV genotyping was performed using the Linear Array HPV Genotyping kit (Roche Diagnostics). The details of HPV genotyping and classification of high-risk HPV types has been previously described [23]. HPV status was recorded as positive or negative. HPV52 cannot be specifically detected using the Linear Array HPV Genotyping protocol. The kit contains a cross-reactive probe that detects HPV 33, 35, 52 and 58 combined. Samples that are positive for HPV 33, 35, and/or 58 individually, as well as the cross-reactive probe have an uncertain HPV 52 status. For our study these samples were interpreted as probably positive for HPV 52.

Classification of HPV-risk was based on the epidemiological classification of HPV types associated with cervical cancer [24]. HPV types: 16, 18, 31, 33, 35, 39, 45, 51, 52, 56, 58, 59, 68, 73, 82 are considered high-risk HPV types; HPV types: 26, 53, 66 are considered probable high-risk HPV types and HPV types: 6, 11, 40, 42, 43, 44, 54, 61, 70, 72, 81, CP6108 are considered low-risk HPV types. For this study, the probable high-risk HPV types and high-risk HPV types were grouped together. All other HPV types that have not been classified as high- or low-risk were classified as undetermined risk.

### 2.4. Statistical Analysis

Statistical analysis was performed using STATA 10 (StataCorp LP, College Station, TX). Fisher’s exact tests were performed to test for group differences between oral HPV-positive and negative groups, with oral HPV status defined by a positive result for either mouthwash or oral brush samples. A *p*-value < 0.05 was considered statistically significant and *p* > 0.05 and <0.1 was considered marginally statistically significant. Exact logistic regression analyses were performed in order to identify the predictors of oral HPV infection [25]. The conditional maximum likelihood estimates (CMLEs) are reported and when the parameters for the CMLEs are infinite, median unbiased estimates (MUEs) were reported.

## 3. Results

### 3.1. Characteristics of the Study Population and Factors Related to Oral HPV Infection

There were 118 women included in this study population. Seventy-five (63.6%) were heterosexual (had vaginal sex and/or oral sex with males only), 32 (27.1%) were bisexual (had vaginal sex and had oral sex with females) and 11 (9.3%) were categorized as other (homosexual exposure or unknown exposure). The oral HPV prevalence for the entire study population was 10.2% (12/118) and was not different between heterosexuals and bisexuals (10.7% (8/75) *vs.* 12.5% (4/32), *p* = 0.784). None of the women that were categorized as “other” were positive of oral HPV infection. Table 1 summarizes the study participants’ demographic and key behavioral characteristics according to oral HPV status. Ages ranged from 24–78 years, with a mean age of 51.8 years. There were no statistically significant differences for oral HPV status according to race or age. There was a marginal difference in smoking status based on oral HPV infection (*p* = 0.068). A larger proportion of the oral HPV-positive women were never smokers compared to the oral HPV-negative women (75% (9/12) *vs.* 54% (56/104)). Among the women that were ever smokers, the distribution of former versus current smokers was different according to oral HPV status. The majority of the oral HPV-positive women were current smokers rather than former smokers (2/12, 17% *vs.* 1/12, 8%) while the opposite was observed for the oral HPV-negative women. After adjusting for age, age at first vaginal sex and oral sex exposure, and using never smokers as the reference group, the odds of oral HPV-positivity for current smokers compared to never smokers was not statistically significantly different (Adj OR: 0.7, 95% confidence interval [CI]: 0.1–4.6) while for former smokers, the odds of oral HPV-positivity was marginally statistically significantly lower (Adj OR: 0.1, 95% CI: 0.0–1.1).

Although there was no statistically significant difference in oral HPV infection based on vaginal sex exposure (12/12, 100% *vs.* 98/104, 94%), the prevalence of oral HPV appears to increase with the number of vaginal sex partners (Table 1). Among the women with a history of vaginal sex (*n* = 110), oral HPV status was statistically significantly different according to oral sex exposure (*p* = 0.039). A higher proportion of oral HPV-positive women reported that they had no history of oral sex exposure compared to oral HPV-negative women (4/12, 33% *vs.* 7/98, 8%). After adjusting for age, age at first vaginal sex and smoking, women who reported a history of vaginal sex but no oral sex exposure were more than six times likely to be oral HPV-positive compared to women who reported a history of vaginal sex and oral sex with males (Adj OR: 6.6, 95% CI: 1.0–43.3). There were no differences in oral HPV infection according to the total number of oral sex partners (data not shown). A higher proportion of women that were oral HPV-positive were over the age of 21 years old when they had their first oral sex exposure compared to the oral HPV-negative women (4/7, 57% *vs.* 42/89, 47%) but this difference was not statistically significant.

Table 2 describes the relationship between oral HPV infection and cervical HPV infection, history of abnormal cervical cytology and history of sexually transmitted diseases including HPV and genital warts. No differences were observed according to oral HPV status except a marginal difference in the proportion of women with a history of HPV infection or genital warts was observed. Twenty-five percent (3/12) oral HPV-positive women had a history of HPV and/or genital warts compared to 9% (10/106) of oral HPV-negative women (*p* = 0.104).

**Figure 1:**
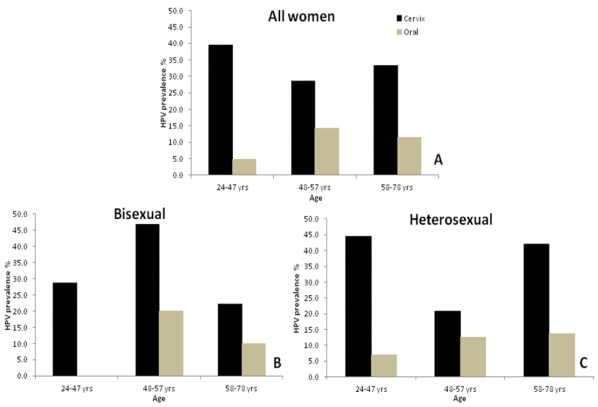
Prevalence of oral and cervical HPV infection according age and type of sexual exposure (**A**) all women (**B**) Bisexual (**C**) Hetersexual.

For all of the women combined, irrespective of sexual exposure, the prevalence of oral HPV infection appears to vary inversely with age when compared to cervical HPV infection (Figure 1A). As expected, cervical HPV infection was highest for women 24–47 years and appears to decrease as age increases. In contrast oral HPV infection was lowest in women 24–47 years and appears to increase as age increases. A similar trend was observed for heterosexual women (Figure 1C) but for bisexual women, cervical HPV infection was lower for younger women and highest for women ages 48–57 years (Figure 1B). Oral HPV infection in bisexual women also was highest for women ages 48–57 years. All other women (homosexuals, virgins and women with incomplete sexual exposure data) were not oral HPV-positive, and while cervical HPV infection was detected in the younger women ages 24–47 (data not shown).

### 3.2. Cervical HPV and Oral HPV Infections

Among the 12 women found to be infected with oral HPV, five women (42%) had at least one known or probable high risk HPV type, 5 (42%) women had HPV types of unknown risk and 2 (16%) women had low risk HPV infections. Three of the twelve women (25%) with oral HPV infection had more than one HPV types present. One woman was infected with high-risk HPV types 16 and 33, the other with high-risk HPV types 35 and 52 and the third was infected with high-risk HPV66 and low-risk HPV70.

In contrast to oral HPV infections, 33.6% (37/110) of the women had detectable cervical HPV infections and among these, more than half of them (20/37, 54%) were infected with at least one known or probable high-risk HPV type and (18/37, 49%) were infected with multiple HPV types. The prevalence of cervical HPV infection did not vary between heterosexuals and bisexuals (35.7% (25/70) *vs.* 35.5% (11/31), *p*-value 0.411) and for all other women the cervical HPV prevalence was significantly lower (11.1%, 1/9). The frequency of high risk types detected in the cervical mucosa ranked from highest to lowest were 52, 33, 56, 35, 59, 68, 16, 18, 39, 45, 51 and 58. The mean age of women with cervical HPV infection was lower (49 years old; range = 24–67 years) than the mean age of women with oral HPV infection (53 years old; range = 37–66 years.) but was not statistically significantly different (*p* = 0.257).

Table 3 summarizes the HPV types detected in both the cervical and oral sites for the women that were oral HPV-positive. There were no similarities in the HPV types detected in the oral and cervical mucosa except that one woman who had HPV52 infections in both sites (Table 3).

## 4. Discussion

HPV is found to be associated with cancer in the oropharynx [26], therefore the relevance of defining risk behaviors related to oral HPV infection in healthy populations may help to identify persons with an increased risk of oral HPV infection. This information would be beneficial and could potentially lead to the implementation of targeted public health interventions that would reduce the likelihood of oral HPV infection in the healthy population. Although HPV-related oral tumors occur in both males and females, only a few studies have investigated the prevalence of oral HPV infection in women in order to identify key sexual behavior factors that could be related to oral HPV infection in this population. The prevalence of oral HPV infection in our study population was low and is consistent with what has been previously reported in the normal oral mucosa [12,19,27,28]. Although participants provided self-defined sexual orientation data, this classification did not correlate with sexual behavior. Women who described themselves as homosexuals also reported a history of sexual involvement with males. Therefore to better categorize the participants according to sexual behavior, we redefined sexual orientation based on the combination of both vaginal and oral sex exposure.

In our study population, there was no significant difference in the prevalence of HPV infection between heterosexual and bisexual women (10.7% (8/75) *vs.* 12.5% (4/32)). In contrast, there was no oral HPV detected among homosexual women, virgins or among women where sexual exposure was unknown. This suggests that oral HPV infection may be more likely associated with sexual exposure to male partners rather than females and further studies are needed to confirm this finding. When oral sex behavior was compared among women who had vaginal sex exposure, there seemed to be an increased prevalence of oral HPV infection for the women that had vaginal sex exposure but no oral sex exposure. This also suggests that that vaginal sex exposure may be a stronger predictor for oral HPV infection. This is supported by results from a recent meta-analysis by Termine, *et al.* The study involved 100 women and reported that the only significant predictor of oral HPV infection was a younger age at first sexual intercourse and no association was found for other types of sexual behavior such as oral and genital sex and the presence of HPV-related cervical lesions [19]. Considering the small number of non-virgin women that reported no oral sex exposure in our study population (*n* = 11), these findings should be interpreted with caution and will need to be confirmed in future studies with a much larger sample size.

Previous studies have found an association between oral HPV status and number of vaginal sex partners, oral sex partners, and kissing partners [5,10]. In our study, we did not evaluate the number of kissing partners but we also observed an increasing trend of positive oral HPV infections as the number of lifetime vaginal sex partners increased. In addition, a history of genital HPV infection or warts in our study seems to contribute to positive oral HPV infections. The lifetime number of sexual partners may not be the best measure of recent sexual activity since it is not yet clear from these data the relationship between timing of oral sex exposure (recent rather than past) and oral HPV status. Therefore age at first vaginal sex exposure as well as age at first oral sex may be more informative. We evaluated the relationship between age at first oral sex exposure and oral HPV status and observed that a slightly higher proportion of women who were older than 21 years at the time of their first oral sex exposure were oral HPV-positive but this was not statistically significantly different from the oral HPV-negative women possibly due to our small sample size. To address the limitations in this current study design, a longitudinal study that includes the evaluation of age at first sexual exposure, recent sexual exposure, HPV infection and persistence might provide new insights into the risk of oral HPV infection.

We observe a statistically significant difference in smoking status among those women who were oral HPV-positive compared to those who were oral HPV-negative. From our results never smokers as well as current smokers may be more likely to be oral HPV-positive. This observation was marginally statistically significant even after adjusting for age and sexual behavior factors. Vacarella, *et al.* reported that the current smoking was associated with the prevalence of cervical HPV infection while there was no association observed for former smokers [29] and similar findings have been reported by others [30,31]. These studies of have shown the association of cervical HPV prevalence with cigarette smoking while studies of HPV in head and neck cancer have reported HPV-positive tumors tend to be located in the oropharynx in nonsmokers yet HPV-positive head and neck tumors are also detected in smokers as well. Therefore further investigation and interpretation of these findings in larger studies are warranted.

A recent study of the natural history of oral HPV reported that persons were significantly more likely to be infected with HPV when their spouse had a persistent infections lasting for the 2 year duration of the study [13]. In our study we attempted to collect information about the history of HPV infection among the sexual partner(s) of the participants in this study but this information was incomplete and could not be evaluated due to this limitation in our study design. We have also considered that the low response rate in this study is also another limitation. Of the 1043 women that were invited to participate, only 88 agreed, suggesting that the population may not be representative of the original pool of potential participants. In an attempt to improve our small sample size, 30 additional women were recruited during routine gynecologic clinic visits at the same institution. While the small sample size limited our analyses, with this consideration, exact conditional logistic regression analyses were appropriately used [25] to calculate odds ratios for possible predictors of oral HPV infection. For small samples, exact regression analysis produces more accurate inference because the calculation does not depend on asymptotic results. Furthermore, exact logistic regression is better able to handle one-way causation, e.g., scenarios where all of the participants are observed to have a positive outcome. Since the confidence intervals for exact logistic regression models are calculated from the exact conditional distributions and are asymmetrical about the estimate, they tend to be wider than the normal-based confidence intervals derived from logistic regression models.

## 5. Conclusions

Overall our findings suggest that sexual behavior and smoking status may be contributing factors that are related to oral HPV infection in women. It is also possible that in women, sexual exposure to male partners may be more predictive of oral HPV infection rather than sexual exposure to female partners but further studies with larger sample size and with a study design that addresses the limitations and lessons learned from this current study are needed to confirm these findings.

## Figures and Tables

**Table 1:** Population characteristics and lifestyle factors according to oral HPV status.

	Oral HPV-Negative *N* = 106 *N* (%)	Oral HPV-Positive *N* = 12 *N* (%)	*p*-Value †
Race			
White	63 (59.4)	8 (66.7)	0.612
Black	37 (34.9)	3 (25.0)	
Other	5 (4.7)	1 (8.3)	
Unknown	1 (0.9)	0 (0.0)	
Age			
<53 years	54 (50.9)	7 (58.3)	0.764
53+ years	52 (49.1)	5 (41.7)	
Smoking status §			
Never smoker	56 (53.9)	9 (75.0)	0.068
Former smoker	39 (37.5)	1 (8.3)	
Current smoker	9 (8.7)	2 (16.7)	
Ever engaged in vaginal sex (*n* = 116) §			
Yes	98 (94.2)	12 (100.0)	1.000
No	6 (5.8)	0 (0.0)	
Lifetime number of vaginal sex partners (*n* = 108) §			
1	20 (20.6)	1 (9.0)	0.438
2–5	51 (52.6)	5 (45.5)	
6+	26 (26.8)	5 (45.5)	
Lifetime number of oral sex partners (male and/or female) (*n* = 101) *			
1	21 (22.6)	1 (12.5)	0.682
2–5	72 (77.4)	7 (87.5)	
Age at first oral sex exposure (Male and/or Female) (*n* = 96) **			
≤21 years	47 (52.8)	3 (42.9)	0.707
>21 years	42 (47.2)	4 (57.1)	
Oral sex exposure among non-virgins (*n* = 106) ¥			
oral sex male exposure θ	78 (83.0)	7 (58.3)	0.039
oral sex female only exposure	9 (9.6)	1 (8.3)	
No oral sex exposure	7 (7.5)	4 (33.3)	

† Fisher’s exact test;

* one woman was excluded due to missing information;

§ two women were excluded due to missing information;

¥ four women were excluded that reported no to history of oral sex with males but history of oral sex with females was unknown;

** five women excluded sue to missing data;

θ includes women with oral sex exposure to males and females, oral sex exposure to males only or oral sex exposure to females was unknown

**Table 2:** Oral HPV status and other clinical factors.

	Oral HPV-Negative *N* = 106 *N* (%)	Oral HPV-Positive *N* = 12 *N* (%)	*p*-Value ^†^

Cervical HPV			
Not Present	67 (63.2)	6 (50.0)	0.525
Present	32 (30.2)	5 (41.7)	
Not done	7 (6.6)	1 (8.3)	
History of abnormal cervical cytology (109) #			
No	67 (67.7)	8 (80.0)	0.721
Yes	32 (32.3)	2 (20.0)	
History of STD Diagnosis §			
No	73(68.9)	9 (75.0)	0.504
Yes	29 (27.4)	2 (16.7)	
Not sure/Unknown	4 (3.8)	1 (8.3)	
History of HPV/genital warts			
No	93 (87.7)	8 (66.7)	0.104
Yes	10 (9.4)	3 (25.0)	
Not sure/Unknown	3 (2.8)	1 (8.3)	

# 9 women were excluded were missing questionnaire data history of abnormal cervical cytology (*i.e.*, greater than 12 months prior to study enrollment);

§ History of being diagnosed with a sexually transmitted disease, such as herpes, chlamydia, gonorrhea, syphilis, trichomonas and HIV infection.

**Table 3:** Cervical HPV infection in oral HPV-positive women

HPV Type (oral)	HPV Type (cervix)
35, (52)	Negative
16, 33	--
52	40, 52, 84
54	Negative
61	83
66,70	84, CP6108
82	Negative
83	61, 62
83	Negative
84	61
84	Negative
84	Negative

(52) = cannot rule out the presence of HPV52; -- Not done.
